# Antioxidant and Anti-Inflammatory Properties of Rubber Seed Oil in Lipopolysaccharide-Induced RAW 267.4 Macrophages

**DOI:** 10.3390/nu14071349

**Published:** 2022-03-24

**Authors:** Jing Liu, Lulu Zhao, Hongying Cai, Zitao Zhao, Yongbao Wu, Zhiguo Wen, Peilong Yang

**Affiliations:** Institute of Feed Research, Chinese Academy of Agricultural Sciences, Beijing 100081, China; liujing2615@163.com (J.L.); zhll2014@yeah.net (L.Z.); hongyingcai1986@126.com (H.C.); 82101205296@caas.cn (Z.Z.); woblin@163.com (Y.W.)

**Keywords:** rubber seed oil, antioxidant, anti-inflammatory, Nrf2 signal pathway, NF-*κ*B signal pathway

## Abstract

Rubber seed oil (RSO) is a typical PUFA-enriched plant oil, but it has not been widely used as a healthy edible oil resource due to the lack of understanding of its nutritional values, health biological effects, and action mechanisms. This work was conducted to characterize the basic physicochemical properties, evaluate the antioxidant and anti-inflammatory properties, and explore the involved mechanisms of RSO in LPS-induced RAW 264.7 cells. In the present study, the basic physicochemical parameters of RSO indicated that RSO has good qualities as a potential edible plant oil resource. In LPS-induced macrophages, RSO supplementation displayed a significant antioxidant effect by decreasing ROS and MDA levels as well as elevating T-AOC. In addition, RSO supplementation showed an anti-inflammatory effect by reducing the production of NO, IL-1*β*, IL-6, and TNF-*α* while promoting the production of IL-10. Moreover, RSO supplementation decreased the mRNA expression of *IL-6*, *IL-1β*, *TNF-α*, *iNOS*, and *MCP-1* genes while increasing the mRNA expression of the *IL-10* gene. Furthermore, RSO supplementation increased Nrf2 protein expression and up-regulated antioxidant genes (*HO-1* and *NQO-1*), which was accompanied by the decrease in TLR4 protein expression and NF-*κ*B p65 phosphorylation as well as I*κ*B*α* phosphorylation. This study provided some insight into the applications of RSO as a healthy edible oil resource.

## 1. Introduction

Rubber seeds are one of the main by-products of natural rubber plantations, which are very rich in oil, with 40–50% oil content of the seed kernel [1,2]. As reported, there is a potential production of 1.94 million tons of rubber seed oil (RSO) in Southeast Asia, which is the main plantation area of rubber trees [3]. In the past, RSO was mainly used in industrial products, such as biodiesel [1], surface coatings [4], and soap ingredients [5]. In recent years, RSO as a plant lipid source has been added to diets in laying hens [6], cows [7], and fish [8], which could regulate the composition of fatty acids and has biological functions. In fact, in some folk areas, RSO has been used as edible oil. However, as a potential renewable edible oil resource, research on the physical and chemical properties of RSO, especially its potentially beneficial biological functions, is still limited and needs to be further studied.

RSO does not contain any unusual fatty acids, mainly including palmitic acid (C16:0), stearic acid (C18:0), oleic acid (C18:1 n-9), linolenic acid (ALA, C18:3 n-3), and linoleic acid (LA, C18:2 n-6) [9]. It has been reported that RSO contains more than 50% polyunsaturated fatty acids (PUFA) [10]. Our previous study showed that RSO was very rich in LA and ALA, which amount to 55.65% of the total fatty acids [6], indicating that RSO is a typical PUFA-enriched plant oil. In addition, we calculated the n-6/n-3 PUFA ratio of RSO to be 1.92, indicating that RSO has a balanced n-6/n-3 PUFA ratio (2:1 to 5:1, recommended by FAO). Dietary PUFA interferes with a variety of physiological and pathophysiological processes, thereby affecting health as well as disease processes [11,12]. It is well known that PUFA is beneficial against cardiovascular disease, cancer, oxidative stress, and inflammation [13,14].

A growing body of reports supports the therapeutic potential of PUFA on diseases based on its antioxidant and anti-inflammatory properties [13,15]. Both n-3 and n-6 PUFAs play important roles in antioxidation and anti-inflammation. Previous studies found that n-3 PUFA (ALA, DHA, EPA) and n-6 PUFA (AA, LA) inhibited reactive oxygen species (ROS) and reactive nitrogen species (RNS) formation and decreased the expression of *iNOS* in LPS-induced macrophages [16]. The reported antioxidant and anti-inflammatory properties of PUFA were partly related to the increase in endogenous antioxidant enzyme activities, the block of pro-inflammatory cytokines production, the decrease in *COX-2*, *iNOS*, *IL-1β*, *IL-6*, and *TNF-α* relative mRNA expression, the activation of the nuclear factor erythroid 2-related factor 2 (Nrf2) pathway, and the inhibition of nuclear factor kappa-light-chain-enhancer of activated B cells (NF-*κ*B) signaling [17,18,19]. Moreover, studies have shown that the balanced ratio of dietary n-6/n-3 PUFA is beneficial to human health and is associated with relatively lower levels of inflammation and stronger resistance to oxidative stress [20,21]. From the perspective of the fatty acids profile of RSO, we speculated that RSO, as a kind of PUFA-enriched plant oil, has the potential to exert antioxidant and anti-inflammatory effects as an edible oil with health benefits. Although some preliminary studies have reported that RSO has shown DPPH and ABTS radical scavenging abilities [22], the potential of hypolipidemic foods [23], and the effect of reducing plasma TNF-*α* and INF-*γ* concentrations [7], there are still few studies on its antioxidant and anti-inflammatory biological activities and the relevant action mechanisms, resulting in limited application of RSO in the food and pharmaceutical fields.

To promote the application of RSO as a healthy edible oil and clarify its potential antioxidant and anti-inflammatory biological properties, the present work characterized the basic physicochemical properties, evaluated the antioxidant and anti-inflammatory properties, and explored the underlying action mechanisms of RSO in LPS-induced macrophages. The finding of this study could provide some insight into the application of RSO as a healthy functional edible oil resource in food fields.

## 2. Materials and Methods

### 2.1. Reagents

Lipopolysaccharides (LPSs, from Escherichia coli serotype 026: B6) were from Sigma Chemical Co. (St. Louis, MO, USA). The CCK-8 Kit, ROS Assay Kit, T-AOC Assay Kit, Lipid Peroxidation MDA Assay Kit, Griess Reagent, ELISA kits for TNF-*α*, IL-1*β*, IL-6, and IL-10 were from Beyotime Biotechnology Co. (Shanghai, China). Anti-TLR4 antibody (ab13556), anti-NF-*κ*B p65 antibody (ab32536), anti-NF-*κ*B p65 (phospho S536) antibody (ab76302), anti-beta actin antibody (ab8227), and secondary antibody (ab97051) were from Abcam (Cambridge, MA, USA). Human/Mouse/Rat Keap1 antibody (MAB3024) and Human/Mouse Nrf2 antibody (AF3925) were from R&D Systems (Minneapolis, MI, USA). I*κ*B*α* (44D4) Rabbit mAb and Phospho-I*κ*B*α* (Ser32) (14D4) Rabbit mAb were from Cell Signaling Technology (Beverly, MA, USA).

### 2.2. Characterization of Physicochemical Properties of RSO

RSO was obtained by the supercritical CO_2_ extraction of rubber seeds, and the fatty acids profile of RSO was analyzed by GC-MS in our previous study [6]. The color measurement of RSO was evaluated by digital color image analysis (DigiEye System, VeriVide Ltd., Leicester, UK), and hunter color parameters were presented as L*, a*, and b* values. The refractive index of RSO was detected using an Abbé refractometer (Atago U.S.A., Inc., Bellevue, WA, USA). The acid values, iodine values, peroxide values, saponification values, and unsaponifiable matter contents of RSO were determined by standard methods according to the recommendations of the American Oil Chemist’s Society [24]. The total cyanide content of RSO was detected using a colorimetric method with modification by spectrophotometry (Thermo Fisher Scientific, Vantaa, Finland) at 638 nm [25]. Mycotoxins, including deoxynivalenol (DON), aflatoxin B1 (AFB1), and zearalenone (ZEA), were determined by HPLC-MS, and the results were expressed in microgram or milligram per kilogram oil. The total phenolic content (TPC) of RSO was determined using the Folin-Ciocalteu method, and the total flavonoids content (TFC) of RSO was measured by the aluminum trichloride colorimetric method [26]. TPC and TFC were estimated based on a standard calibration curve of gallic acid and rutin, respectively. Experiments were carried out in triplicate.

### 2.3. Cell Culture and Cell Viability Testing

The RAW 264.7 cells were cultured in DMEM medium supplementing 10% FBS, 100 Units/mL penicillin, and 100 μg/mL streptomycin. The cells were cultured in a humidified atmosphere at 37 °C containing 5% CO_2_ and were sub-cultured every two or three days to maintain an exponential growth phase during experiments. RSO was sterilized by filtration with a 0.2 μm membrane and dissolved in DMSO at a final concentration of no more than 1‰ in all assays.

In the cell viability testing, cells were inoculated at the 96-well plate with a density of 1 × 10^5^ and cultured for 3 h until the cells adhered to the wall. Then, the cells were treated with a range of concentrations (0, 12.5, 25, 50, 100, 150, and 200 μg/mL) of RSO for 24 h. Cell viability was determined with the manufacturer’s protocol of the CCK-8 Kit (Beyotime Biotechnology Co., Shanghai, Beijing). The absorbance (A) was measured by the spectrophotometer (Thermo Fisher Scientific, Vantaa, Finland) at 450 nm. In this study, cell survival rate (%) = A (treatment group)/A (the control group) × 100%.

### 2.4. Determination of Intracellular ROS Level

The levels of intracellular ROS were analyzed using a ROS Assay Kit (Beyotime Biotechnology Co., Shanghai, Beijing). Briefly, macrophage cells were seeded in 96-well black plate and grew to the confluence of about 90%. Next, the cells were exposed to RSO (0, 50, 100, and 150 μg/mL) and LPS (l μg/mL). After 12 h, the cells were incubated with DCFH-DA for 20 min at 37 °C. Then, we discarded the supernatant, washed the plate 3 times with PBS, resuspended cells with 100 μL PBS, and measured them with a microplate reader (Tecan, Switzerland).

### 2.5. Determination of NO, MDA, and T-AOC

The RAW 264.7 cells were seeded in a 24-well plate with a density of 1 × 10^6^/mL until the cells grew to the confluence of about 90%. Cells were treated by a range of concentrations of RSO (0, 50, 100, and 150 μg/mL) and LPS (l μg/mL) for 24 h. Then, the culture medium was collected to determine the NO concentration, and cell lysate was obtained by RIPA tissue/cell lysis buffer to detect T-AOC and MDA levels. The protein content of cell lysate was determined by the BCA protein assay kit (Beyotime Biotechnology Co., Shanghai, Beijing).

NO concentration was measured by Griess reagent (Beyotime Biotechnology Co., Shanghai, Beijing). The culture supernatant was added to a 96-well plate, followed by Griess reagent according to the recommendation. Then, the plate was allowed to stand for 5 min, and the absorbance was detected by a spectrophotometer at 540 nm. The concentration of NO was calculated based on a NaNO₂ standard curve (dissolved in DMEM with 10% FBS). T-AOC assay was carried out according to the Ferric-Reducing Ability of Plasma (FRAP) method by a T-AOC Assay Kit (Beyotime Biotechnology Co., Shanghai, Beijing). The principle of the FRAP method is that under acidic conditions, the antioxidants can reduce ferric-tripyridyltriazine (Fe^3+^-TPTZ) to produce blue Fe^2+^-TPTZ and then determine the blue Fe^2+^-TPTZ at 593 nm to obtain the T-AOC of each sample. The T-AOC of samples was expressed by the concentration of FeSO_4_ standard solution (millimole per gram protein). A colorimetric method based on MDA and the thiobarbituric acid reaction was used to estimate cellular MDA levels by the MDA Assay Kit. MDA concentration was measured by the manufacturer’s protocol, and the MDA levels were expressed as micromole per gram protein (μM/g). All the experiments were independently repeated 3 times.

### 2.6. Determination of Inflammatory Cytokine

When RAW 264.7 cells grow to the confluence of about 90% in the 24-well plate, the cells were treated by RSO and LPS as Section 2.5 for 24 h. Then, the culture medium was obtained and stored at −20 °C for further detection. Levels of IL-1*β*, IL-6, IL-10, and TNF-*α* were determined by ELISA. ELISA assays were performed with commercial kits and determined by spectrophotometry (Thermo Fisher Scientific, Vantaa, Finland) at 450 nm with 3 repeats independently. Concentrations of inflammatory cytokines were calculated by standard curves and expressed as microgram or nanogram per milliliter.

### 2.7. RNA Extraction and qRT-PCR

The RAW 264.7 cells were treated by RSO (0, 50, 100, and 150 μg/mL) and 1 μg/mL LPS for 12 h. We discarded the medium and washed the plate with PBS 3 times. Then, the total RNA was extracted from cells with TRIzol^®^ reagent (Invitrogen, Thermo Fisher Scientific, Reinach, Switzerland). Next, chloroform was added to the sample tubes, and the total RNA was collected in the aqueous phase after centrifugation. Finally, the total RNA was precipitated by isopropanol, washed with 75% ethanol, and redissolved in RNAse-free water. The concentration and purity of the total RNA were determined using a spectrophotometer (Thermo Fisher Scientific, Vantaa, Finland). To obtain cDNA, 500 ng of RNA was used for reverse transcription using the TransScript First-Strand cDNA Synthesis SuperMix (TransGen Biotech, Beijing, China) at the following conditions: 15 min at 42 °C, 15 s at 85 °C. The cDNA was stored at −20 °C for further experiments. The quantitative real-time PCR was performed using a *Power* SYBR^®^ Green PCR Master Mix kit (Applied Biosystems, Thermo Fisher Scientific, Finland) by a CFX96™ Real-Time System (Bio-Rad, Hercules, CA, USA). Based on the threshold cycle (Ct) values of the target genes and internal control gene, the relative expression level of target genes in each group were calculated using the 2^−ΔΔCt^ method, and results were analyzed in triplicate assays. Primers of detected genes are listed in Supplementary Table S1.

### 2.8. Western Blotting

RAW 264.7 cells were harvested and lysed using RIPA buffer with protease and phosphatase inhibitors for 30 min to extract the total proteins. The total protein was determined by the BCA protein assay kit. Total proteins were separated by SDS-PAGE and transferred onto a polyvinylidene fluoride (PVDF) membrane. The PVDF membrane was blocked with 5% (*w*/*v*) nonfat dry milk or bovine albumin for 2 h and incubated with specific primary antibodies (1:1000 dilution) overnight at 4 °C. Then, the membrane was incubated with HRP-conjugated secondary antibody (1:5000 dilution) for 3 h. Next, the membrane was incubated with Pierce™ ECL Western Blotting Substrate (Thermo Fisher Scientific Inc., Waltham, MA, USA) for another 10 min. Band intensity was measured using the ChemiDoc XRS+ chemiluminescence image system (Bio-Rad, Hercules, CA, USA) and quantified using Image J gel analysis software. All Western blotting analyses of this study were performed in triplicate.

### 2.9. Statistical Analysis

SPSS 18.0 software was used for statistical analysis. In this study, data were expressed as mean ± SD. One-way analysis of variance (ANOVA) and Student’s-test (*t*-test) were used to evaluate the significant differences among groups. Asterisks (* *p* < 0.05, ** *p* < 0.01) indicated significant differences from the normal control group, and pound signs (^#^ *p* < 0.05, ^##^ *p* < 0.01) indicated significant differences from the LPS-induced group.

## 3. Results

### 3.1. Physicochemical Characteristics of RSO

Physical properties (color and refractive index) and chemical properties (acid value, iodine value, peroxide value, and saponification value) of RSO were determined to characterize this oil, and the physicochemical parameters of RSO are given in Table 1. In this study, the CIE L*, a*, and b* color parameters of RSO were 87.72, 2.28, and 31.70, respectively. The refractive index of RSO was 1.4697, which is similar to those reported by previous research [27]. The acid, iodine, peroxide, saponification values, and unsaponifiable matter content of RSO were 0.75 mg KOH/g oil, 137.51 g iodine/100 g oil, 3.12 mmol/kg oil, 194.35 mg KOH/g oil, and 1.02%, respectively. The TPC of RSO was 1032.60 mg GAE/kg oil (standard curve equation y = 0.0096x + 0.0776, R^2^ = 0.9998), and the TFC of RSO was 304.31 mg/kg (y = 0.0006x + 0.0871, R^2^ = 0.9939). In the present study, no cyanide was detected by the colorimetric method. Moreover, three of the most widely spread classes of mycotoxins, including deoxynivalenol (DON), aflatoxin B1 (AFB1), and zearalenone (ZEA), were detected here by HPLC-MS. The contents of DON and ZEA were 0.8 μg/kg and 0.64 mg/kg, respectively. The content of AFB1 was less than 0.6 μg/kg. The contents of all these three mycotoxin levels of RSO were lower than the recommendation by the FDA/WHO [28]. Our previous study revealed the fatty acid profile of RSO [6], which was also summarized in Table 1. The fatty acids of RSO were mainly unsaturated. Monounsaturated fatty acids of RSO were mainly oleic acid (C18:1n-9), with a content of 25.79%. The total content of PUFA of RSO was 56.88%, which were mainly linoleic acids (LA, C18:2n-6) and linolenic acids (ALA, C18:3n-3), accounting for 37.26% and 19.43%, respectively. The combination of the above findings suggested that the RSO was typical PUFA-enriched plant oil and possessed the desirable qualities of edible plant oil.

### 3.2. Cytotoxicity of RSO on RAW 264.7 Cells

**Figure 1:**
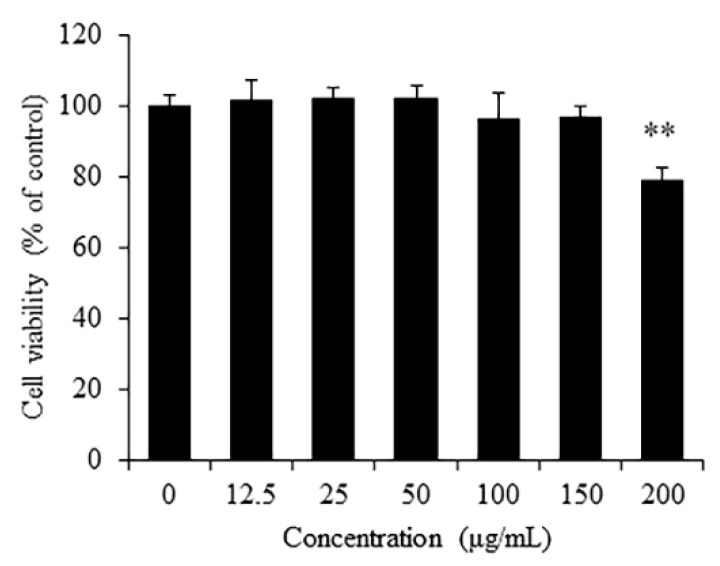
Cytotoxicity of RSO on RAW 264.7 cells. Cells were treated by RSO (0, 12.5, 25, 50, 100, 150, and 200 μg/mL) for 24 h, *n* = 6. ** *p* < 0.01 vs control group.

In this study, we treated RAW 264.7 cells with a range of concentrations of RSO (0, 12.5, 25, 50, 100, 150, and 200 μg/mL) to determine the safe concentration. The results showed that when the concentration of RSO was less than or equal to 150 μg/mL, it did not have any cytotoxic effect on RAW 264.7 cells (Figure 1). However, a marked decrease (*p* < 0.01) in cell viability (79.02% of control) was observed when the RSO concentration was raised to 200 μg/mL. In the current results, non-cytotoxic concentrations of RSO in RAW 264.7 cells were observed at 12.5–150 μg/mL. Thus, in subsequent experiments to evaluate the antioxidant and anti-inflammatory effects of RSO, treatment concentrations of 50 to 150 μg/mL were used in RAW 264.7 cells. Previous studies reported that no cytotoxicity of RSO was observed in B16-F10 melanoma cells and 3T3-L1 cells at 0.1–100 μg/mL [29]. The observed non-cytotoxic concentrations of RSO to cells may be related to the concentration and combination of the fatty acid, especially PUFA.

### 3.3. Antioxidant Properties of RSO

#### 3.3.1. Effect of RSO on ROS Levels of LPS-Induced RAW 264.7 Cells

**Figure 2:**
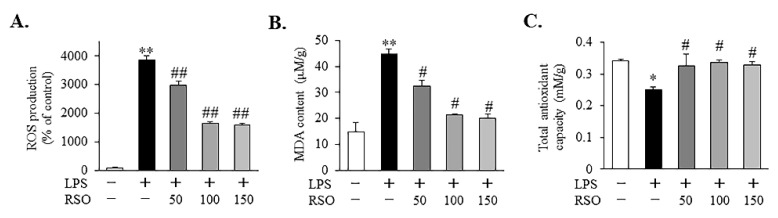
Effect of RSO on ROS, MDA, and T-AOC of LPS-induced RAW 264.7 cells. (**A**) ROS levels of each group, *n* = 6; (**B**) MDA levels of each group, *n* = 3; (**C**) Total antioxidant capacity (T-AOC) of each group, *n* = 3. * *p* < 0.05, ** *p* < 0.01 vs control group; ^#^ *p* < 0.05, ^##^ *p* < 0.01 vs LPS-induced group.

Reactive oxygen species (ROS), as one of the most critical biomarkers of oxidative stress, reflect the effects of the antioxidant in controlling the extent of oxidation. In this work, the effect of RSO on ROS production in LPS-induced macrophage cells was investigated. From Figure 2A, compared with the control group, the concentration of ROS in the LPS-induced group was significantly increased to 38.61-fold, indicating that 1 μg/mL of LPS could successfully establish the oxidative stress model of RAW 264.7 macrophage cells. Compared with LPS-induced macrophages, RSO treatment significantly reduced the generation of ROS dose-dependently (Figure 2A, *p* < 0.05). The ROS production was decreased by almost 60% with the treatment of the 150 μg/mL RSO, which indicated that RSO had a significant antioxidant effect in LPS-induced macrophages. Similar to our results, both LA and ALA, the major PUFA of RSO, have been reported to exert significant antioxidant properties by inhibiting ROS formation in LPS-induced macrophages [16].

#### 3.3.2. Effect of RSO on MDA Levels of LPS-Induced RAW 264.7 Cells

MDA is one of the natural products of lipid peroxidation when cells undergo oxidative stress, so the level of lipid peroxidation could be reflected by detecting MDA content. The MDA contents of each group are shown in Figure 2B. Our results revealed that the concentration of MDA increased significantly when RAW 264.7 cells were exposed to LPS, where the MDA level was about 3.04-fold higher than that of the control (*p* < 0.01). Compared with the LPS-induced group, significant drops of MDA levels were observed dose-dependently in the RSO treatment groups (*p* < 0.05). The observed reduction in MDA levels could be linked to the reduction in LPS-induced ROS production by RSO treatment.

#### 3.3.3. Effect of RSO on T-AOC of LPS-Induced RAW 264.7 Cells

The total antioxidant capacity (T-AOC) was measured in our study. Compared with control, a dramatic decrease (*p* < 0.05) of T-AOC was observed in cells stimulated with LPS (Figure 2C). Interestingly, we found that RSO could significantly alleviate the reduction in T-AOC of macrophages caused by LPS treatment. No significant differences (*p* > 0.05) were presented among the three treatment groups with different concentrations of RSO in this study. This result was consistent with another report, which revealed the benefits of PUFA (EPA and DHA) treatment on T-AOC in PC12 cells [30]. The T-AOC is a measure of the cumulative effects of the antioxidants present in biological samples, which give more reliable biological information than that of individual antioxidants. These results indicated that RSO showed a significant antioxidant effect in LPS-induced RAW 264.7 cells.

#### 3.3.4. Effect of RSO on Nrf2-Keap1 Pathway of LPS-Induced RAW 264.7 Cells

**Figure 3:**
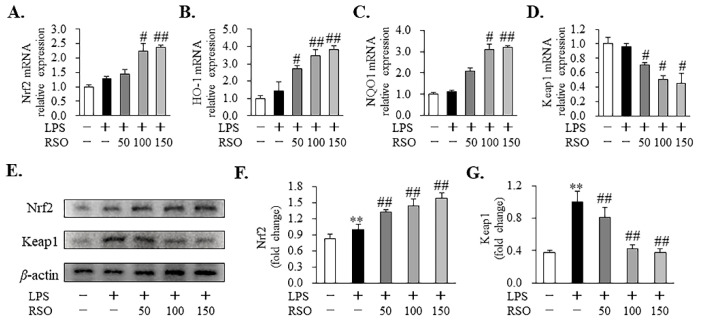
Effect of RSO on Nrf2-Keap1 pathway of LPS-induced RAW 264.7 cells. Relative mRNA expression of *Nrf2* (**A**), *HO-1* (**B**), *NQO1* (**C**), and *Keap1* (**D**) genes normalized to *β*-actin and expressed as fold change of the control group, *n* = 3. (**E**) The protein levels of Nrf2 and Keap1 were analyzed by immunoblotting with *β*-actin as the internal reference protein for normalization, *n* = 3. Relative protein expression of Nrf2 (**F**) and Keap1 (**G**) were expressed as fold change of the LPS-induced group. ** *p* < 0.01 vs control group; ^#^ *p* < 0.05, ^##^ *p* < 0.01 vs LPS-induced group.

In this study, we further studied the antioxidant mechanism and the relative expression of related genes and proteins in the Nrf2 signaling pathway. As shown in Figure 3, RSO supplementation significantly elevated relative mRNA expressions of *Nrf2*, *HO-1*, and *NQO1* genes while inhibiting relative mRNA expression of *Keap1* in LPS-induced cells. In agreement with the effects of RSO on mRNA expression, RSO treatment also affected Nrf2 signaling pathway-related protein expression. Compared with the LPS-induced group, RSO treatment significantly elevated (*p* < 0.01) Nrf2 protein expression, and it markedly decreased (*p* < 0.01) Keap1 protein expression dose-dependently (Figure 3F,G). Consistent with our result, previous reports showed that PUFA and PUFA-enriched oil are beneficial in alleviating oxidative damage through activating the Nrf2/Keap1 signaling pathway [17,31].

### 3.4. Anti-Inflammatory Properties of RSO

#### 3.4.1. Effect of RSO on NO Contents of LPS-Induced RAW 264.7 Cells

**Figure 4:**
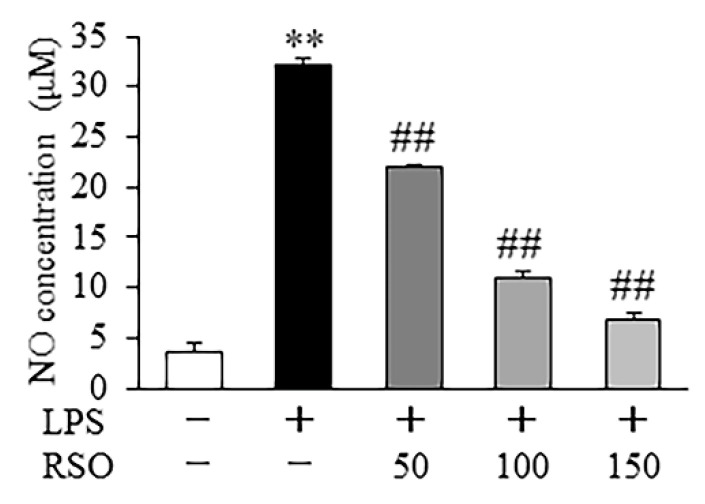
Effect of RSO on NO contents of LPS-induced RAW 264.7 cells. Cells were treated by RSO (0, 50, 100, and 150 μg/mL) and LPS (l μg/mL) for 24 h; then, culture solution was obtained for detection, *n* = 3. ** *p* < 0.01 vs control group; ^##^ *p* < 0.01 vs LPS-induced group.

Nitric oxide (NO) is an important signaling molecule in the pathogenesis of inflammation, which is generally considered as a pro-inflammatory mediator due to overproduction in abnormal situations [32]. From Figure 4, LPS (1 μg/mL), stimulation significantly increased (*p* < 0.01) NO production by about 10-fold compared with the control group. This result suggested that RAW 264.7 cells challenged with 1 μg/mL LPS can also be used to establish an inflammation model. Our result showed that NO concentrations dose-dependently decreased in RSO treatment groups, suggesting that RSO could reduce NO levels in LPS-induced inflammatory RAW 264.7 cells.

#### 3.4.2. Effect of RSO on Inflammatory Cytokine of LPS-Induced RAW 264.7 Cells

**Figure 5:**
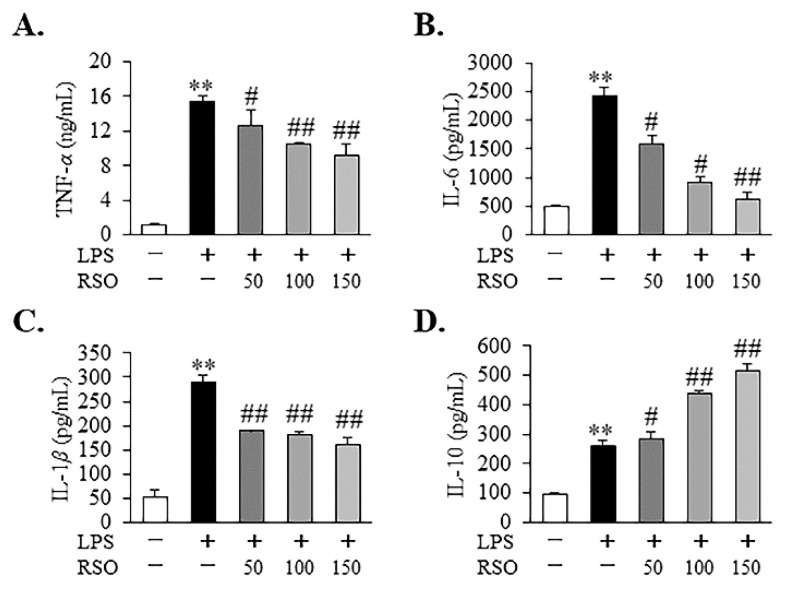
Effect of RSO on inflammatory cytokine content of LPS-induced RAW 264.7 cells. (**A**) TNF-*α* contents of each group; (**B**) IL-6 contents of each group; (**C**) IL-1*β* contents of each group; (**D**) IL-10 contents of each group, *n* = 3. ** *p* < 0.01 vs control group; ^#^ *p* < 0.05, ^##^ *p* < 0.01 vs LPS-induced group.

In this work, levels of inflammatory cytokines were detected to determine the anti-inflammatory properties of RSO in our research. There was a significant trend of increasing the production of TNF-*α*, IL-6, IL-1*β*, and IL-10 in RAW 264.7 cells stimulated by 1 μg/mL LPS, as shown in Figure 5 (*p* < 0.01). In this study, we observed that RSO treatment significantly decreased (*p* < 0.05) TNF-*α* and IL-6 concentrations dose-dependently in LPS-induced cells (Figure 5A,B). The IL-1*β* production was also reduced in the RSO treatment group (Figure 5C); however, there was no significant difference among different concentrations of the RSO treatment group (*p* > 0.05). What is more, RSO treatment further increased IL-10 contents dose-dependently (Figure 5D). Consistently, other studies observed that PUFA could regulate the production of inflammatory cytokines [18,33].

#### 3.4.3. Effect of RSO on Inflammatory-Related Gene Expression of LPS-Induced RAW 264.7 Cells

**Figure 6:**
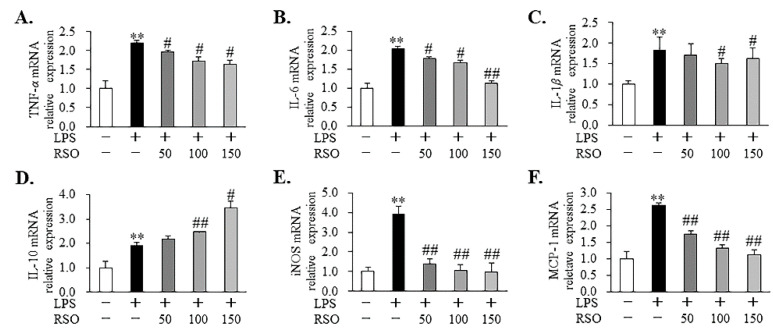
Effect of RSO on inflammatory-related gene relative expression of LPS-induced RAW 264.7 cells. Relative mRNA expression of *TNF-α* (**A**), *IL-6* (**B**), *IL-1β* (**C**), *IL-10* (**D**), *iNOS* (**E**), and *MCP-1* (**F**) genes normalized to *β*-actin and expressed as fold change of the control group, *n* = 3. ** *p* < 0.01 vs control group; ^#^ *p* < 0.05, ^##^ *p* < 0.01 vs LPS-induced group.

As we expected, in LPS-induced RAW 264.7 cells, RSO treatment statistically decreased (*p* < 0.05) relative mRNA expression of *IL-6*, *IL-1β*, and *TNF-α* genes, while it increased the relative mRNA expression of the *IL-10* gene, which was consistent with the production of inflammatory cytokines (Figure 5). In addition, RSO treatment reduced *iNOS* mRNA expression (Figure 6E), which was consistent with the result that RSO treatment reduced NO content. Moreover, the present results showed that RSO treatment also inhibited *MCP-1* mRNA expression dose-dependently in LPS-induced macrophages (Figure 6F). MCP-1 has a vital role in the migration and infiltration process of inflammatory cells by attracting or enhancing the expression of other inflammatory factors [34]. The anti-inflammatory effects of PUFA, by reducing relative mRNA expression of *MCP-1* in vivo and in vitro, were also found in Koto’s research [35].

#### 3.4.4. Effect of RSO on TLR4/NF-*κ*B Pathway of LPS-Induced RAW 264.7 Cells

**Figure 7:**
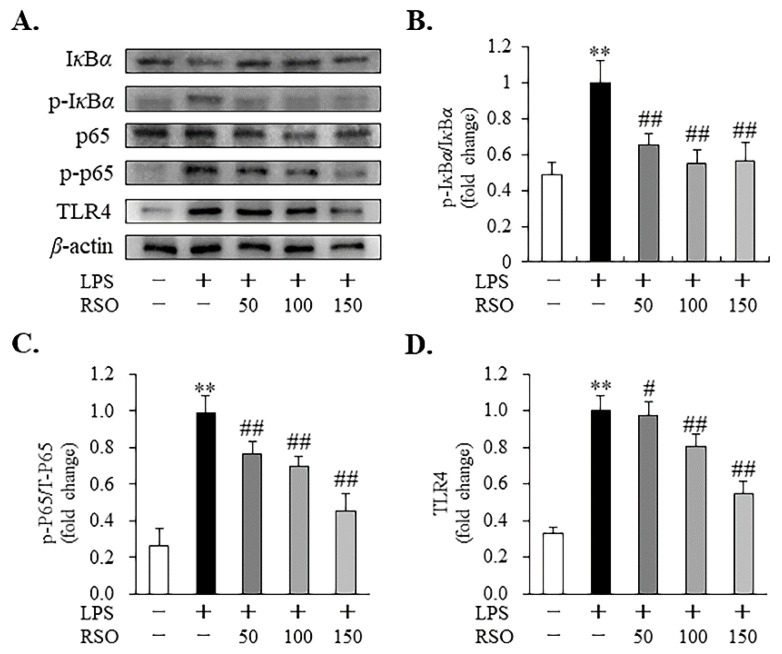
Effect of RSO on the TLR4/NF-*κ*B pathway of LPS-induced RAW 264.7 cells. (**A**) The protein levels of I*κ*B*α*, phosphorylated I*κ*B*α*, total p65, phosphorylated p65, and TLR4 were analyzed by immunoblotting, with *β*-actin as the internal reference protein for normalization. The ratio of phosphorylated I*κ*B*α* to I*κ*B*α* (**B**), the ratio of phosphorylated p65 to total p65 (**C**), and the relative protein expression of TLR4 (**D**) were expressed as fold change of the LPS-induced group, *n* = 3. ** *p* < 0.01 vs control group; ^#^ *p* < 0.05, ^##^ *p* < 0.01 vs LPS-induced group.

To further investigate the anti-inflammatory mechanisms of RSO, the protein expression of TLR4 and levels of phosphorylation of NF-*κ*B p65 and I*κ*B*α* were detected by Western blotting in LPS-induced RAW 264.7 macrophages. From Figure 7, LPS (1 μg/mL) significantly increased (*p* < 0.01) the phosphorylation of I*κ*B*α* and NF-*κ*B p65, while RSO treatment dose-dependently decreased levels of p-I*κ*B*α*/I*κ*B*α* and p-p65/p65 (Figure 7B,C) in LPS-induced cells. In addition, RSO decreased the LPS-induced increase in TLR4 protein expression (Figure 7D). Our results showed that the protein expression of TLR4 and phosphorylation of NF-*κ*B p65 and I*κ*B*α* stimulated by LPS were inhibited by RSO in inflammatory macrophages, indicating that RSO could inhibit the TLR4/NF-*κ*B pathway in LPS-induced RAW 264.7 cells.

## 4. Discussion

PUFA is of the utmost importance for human metabolism and it is attributed to lots of beneficial properties, such as antioxidant, anti-inflammatory, prevention of cardiac diseases, and inhibition of tumor progression [36]. Such beneficial properties are indicative of the nutritional and pharmaceutical potential of dietary PUFA supplementation. Our previous studies have shown that RSO is a typical PUFA-enriched plant oil with a PUFA content of up to 55.65% [6]. Compared with soybean oil, which has an important contribution to dietary PUFA intake in many countries, RSO has a fatty acid composition similar to soybean oil, but its ALA content is 2.86 times higher [4,37]. More importantly, the n-6/n-3 PUFA ratio of RSO was 1.92, which is lower than the n-6/n-3 PUFA ratio in soybean oil of 7.5 [37]. Here, we revealed the basic physicochemical characteristics of RSO, including color, refractive index, acid value, iodine value, peroxide value, and saponification value, indicating the potential of RSO as an edible oil to supplement dietary PUFA. Cyanide is a well-known toxic compound in fresh rubber seeds. However, no cyanide was detected in our experiment. Similarly, Salimon et al. showed that there was no cyanide (C≡N) in RSO by Fourier transform infrared spectroscopy (FTIR) [38]. The total phenolic content (TPC) and the total flavonoid content (TFC) of RSO were 1032.60 mg GAE/kg oil and 304.31 mg/kg, respectively. The TPC of RSO was about twice that of tea seed oil, while the TFC of RSO was similar to that of tea seed oil [26]. To the best of our knowledge, this was the first study of the total polyphenol and total flavonoid contents in RSO. The phenolics and flavonoids present in RSO help the oxidative stability of RSO during storage and may be partly responsible for the anti-inflammatory and antioxidation effects of RSO. In short, based on basic physicochemical properties, PUFA composition, total phenolics, and total flavonoids, it was reasonable to consider RSO as a healthy edible oil resource for providing dietary PUFA.

Reactive oxygen species (ROS), as signaling molecules, play an important role in regulating normal cell physiological functions, but an excessive production of ROS could arise due to oxidative stress, which has been reported to link to the augmented pathogenesis of various diseases, such as diabetes, cancers, atherosclerosis, Alzheimer’s disease, neurodegenerative disorders, cataracts, and inflammation [39,40]. In this study, we found that RSO treatment significantly decreased ROS levels in LPS-induced macrophages (Figure 2). Combined with the results that RSO treatment could significantly reduce MDA content and improve the total antioxidant capacity, we concluded that RSO has antioxidant properties in LPS-induced macrophages. Additionally, this study further revealed the antioxidant mechanism of RSO by detecting the expression of related genes and proteins in the Nrf2 signaling pathway. The Nrf2-Keap1 system is an evolutionarily conserved intracellular defense mechanism to counteract oxidative stress [41]. Under basal conditions, the transcription factor Nrf2 is sequestered in the cytoplasm by Keap1. When the cell is challenged by oxidative stress, Nrf2 detached from Keap1, translocated to the nucleus, heterodimerized with Mafs, and the Nrf2-Maf heterodimer bound to the antioxidant response element (ARE) sequence to induce the expression of antioxidant and metabolic genes [19,41]. In the current work, compared with the LPS-induced group, RSO treatment significantly increased the relative expression of Nrf2 and downstream genes (*HO-1*, *NQO-1*), and it markedly decreased the expression of Keap1 in RAW 264.7 macrophages dose-dependently (Figure 3), which indicated that the antioxidant properties of RSO were related to the activation of the Nrf2/Keap1 pathway in LPS-induced RAW 264.7 cells.

In this work, the anti-inflammatory properties of RSO in LPS-induced RAW 264.7 cells were also evaluated. The results showed that RSO treatment decreased NO contents and inhibited the relative expression of the *iNOS* mRNA dose-dependently. NO production plays a vital role in inflammation [33]. iNOS is the key enzyme that is responsible for most NO synthesis, which is potently induced in the macrophage response to pro-inflammatory stimuli [42]. Apparently, the decreased production of NO was the result of inhibition of *iNOS* mRNA expression in the RSO treatment group. Consistent with our findings, many previous studies have reported that the anti-inflammatory activities of PUFA on macrophages were related to the reduction in NO and *iNOS* mRNA expression [18,32]. Moreover, we found that RSO treatment decreased inflammatory cytokines production, including TNF-*α*, IL-6, and IL-1*β*, while increasing the contents of anti-inflammatory cytokines, such as IL-10. Consistent with our work, previous studies also showed that PUFA-enriched plant oil displayed anti-inflammatory effects by decreasing pro-inflammatory cytokines at mRNA levels and thus regulating inflammatory cytokines production [32,43]. Furthermore, this study revealed that the anti-inflammatory effect of RSO was related to the inhibition of the TLR4/NF-*κ*B pathway. TLR4 is one of the most important cell surface receptors for the natural immune system to recognize pathogenic microorganisms [44]. The stimulation of TLR4 by LPS triggers MyD88-dependent downstream signaling, including NF-*κ*B-dependent pathways, resulting in the expression and the release of critical pro-inflammatory mediators and pro-inflammatory cytokines [45]. A growing body of research revealed the importance of the TLR4/NF-*κ*B pathway in the anti-inflammatory effects of PUFAs, which was consistent with our results. For example, walnut oil, containing 69.0% PUFA and 11.58% ALA, exerted a protective effect on LPS-induced acute intestinal injury in mice by inhibiting the TLR4/NF-*κ*B pathway [43]. DHA- and EPA-enriched Asterias amurensis fatty acids could reduce NF-*κ*B p65 phosphorylation [18]. Our present results indicated that the anti-inflammatory properties of RSO were regulated, at least in part, by suppressing the TLR4/NF-*κ*B pathway in LPS-induced RAW 264.7 cells.

Overall, these findings provided support for the use of RSO as a PUFA-enriched edible oil with anti-inflammatory and antioxidant benefits. To give better play to its nutritional and healthy biological function, further work is required to establish the optimal extraction process of RSO. In addition, more work needs to be performed to remove the unique flavor of RSO to make it more organoleptically acceptable.

## 5. Conclusions

The present work characterized the physicochemical properties of RSO and revealed the significant antioxidant and anti-inflammatory properties of RSO in LPS-induced macrophages. The underlying mechanisms were related to the inhibition of the TLR4/NF-*κ*B signaling pathway and activation of the Nrf2 signaling pathway. Our present findings suggested that the PUFA-enriched RSO had potential health benefits for its antioxidant and anti-inflammatory properties.

## Figures and Tables

**Table 1:** Physicochemical characteristics of RSO.

Physicochemical Parameters ^1^		Fatty Acids Composition (%)	
L*	87.72 ± 0.03	Saturated	
a*	2.28 ± 0.18	Palmitic acid (C16:0)	9.23 ± 1.26
b*	31.70 ± 1.11	Stearic acid (C18:0)	7.49 ± 0.20
Refractive index (n^20^ D)	1.4697 ± 0.0015	Monounsaturated	
Acid value (mg KOH/g)	0.75 ± 0.25	Oleic acid (C18:1 n-9)	25.26 ± 3.62
Iodine value (g iodine/100 g)	137.51 ± 0.72	Polyunsaturated	
Peroxide value (mmol/kg)	3.12 ± 0.05	Linoleic acid (C18:2 n-6)	37.26 ± 3.16
Saponification value (mg KOH/g)	194.35 ± 6.17	Linolenic acid (C18:3 n-3)	19.43 ± 1.59
Unsaponifiable matter (%)	1.02 ± 0.10	Other fatty acids	1.33 ± 3.51
Cyanide	ND ^2^	∑SFA ^3^	17.33 ± 1.31
Aflatoxin B1 (μg/kg)	<0.6	∑MUFA ^3^	25.79 ± 2.23
Zearalenone (mg/kg)	0.64 ± 0.01	∑PUFA ^3^	56.88 ± 1.09
Deoxynivalenol (μg/kg)	0.8 ± 0.1	∑n-3 PUFA	19.48 ± 1.70
TPC ^3^ (mg GAE/kg)	1032.60 ± 21.55	∑n-6 PUFA	37.40 ± 2.79
TFC ^3^ (mg RE/kg)	304.31 ± 3.24	n-6/n-3	1.92 ± 0.31

^1^ Values are mean ± standard deviation in triplicate. ^2^ ND = Not detected. ^3^ TPC, total phenolic content; TFC, total flavonoid content; GAE: gallic acid equivalent; RE: rutin equivalent; SFA, saturated fatty acid; MUFA, monounsaturated fatty acid; PUFA, polyunsaturated fatty acid.

## Data Availability

The datasets used for the current study are available from the corresponding author on reasonable request.

## Supplementary Materials

The following supporting information can be downloaded at: https://www.mdpi.com/article/10.3390/nu14071349/s1, Table S1: Primer sequences.
