# Supplementary material for: Antioxidant and Anti-Inflammatory Properties of Rubber Seed Oil in Lipopolysaccharide-Induced RAW 267.4 Macrophages
Source: Nutrients. 2022 Mar 24;14(7):1349. doi: 10.3390/nu14071349 (PMC9003255; doi:10.3390/nu14071349)
Supplement: Supplementary file 1 [file nutrients-14-01349-s001.zip › nutrients-1637909-supplementary.pdf]

Supplementary Table S1. Primer sequences.

Genes	Type	Sequences
β-actin	Sense	5’-TTGTTACCACCTGGGACG-3’
	Anti-sense	5’-GGCATAGAGCTCTTTACGG-3’
Nrf2	Sense	5’-TCAGCGACAGAAGGACTAAG-3’
	Anti-sense	5’-AGGCATCTTGTTTGGGAATG-3’
Keap-1	Sense	5’-AGTGGCATCCTGCGT TTCT-3’
	Anti-sense	5’-CAACACCACACCAACATTA-3’
HO-1	Sense	5’-TCTGGATGGAGGGAGATACC-3’
	Anti-sense	5’-CAGCAGTCGTGGTCAGTCAA-3’
NQO1	Sense	5’-AGTGGCATCCTGCGTTTCT-3’
	Anti-sense	5’-TCTCCTCCCAGACGGTTTC-3’
iNOS	Sense	5’-AGACTGGATTTGGCTGGTCCCTCC-3’
	Anti-sense	5’-AGAACTGAGGGTACATGCTGGAGCC-3’
IL-6	Sense	5’-GAAGTTCCTCTCTGCAAGAG-3’
	Anti-sense	5’-CGATTTCCCAGAGAACATGTG-3’
IL-10	Sense	5’-GACAACATACTGCTAACCGACTCC-3’
	Anti-sense	5’-GCTCCTTGATTTCTGGGCCATG-3’
IL-1β	Sense	5’-TGCAGAGTTCCCCAACTGGTACATC-3’
	Anti-sense	5’-CATCATCATCCCATGAGTC -3’
TNF-α	Sense	5’-GTCAACCTCCTCTCTGC-3’
	Anti-sense	5’-GCTGGGTAGAGAATGGATG-3’
MCP-1	Sense	5’-GCATCCACGTGTGGCTCA-3’
	Anti-sense	5’-CTCCAGCCTACTCATTGGGATCA-3’
